# Clinical Outcomes in Patients with Schizophrenia Treated with Long-Acting Injectable vs. Oral Antipsychotics: A Naturalistic Study

**DOI:** 10.3390/healthcare13141709

**Published:** 2025-07-16

**Authors:** Francesca Bardi, Lorenzo Moccia, Georgios D. Kotzalidis, Gianluca Boggio, Andrea Brugnami, Greta Sfratta, Delfina Janiri, Gabriele Sani, Alessio Simonetti

**Affiliations:** 1 Department of Neurosciences, Section of Psychiatry, Università Cattolica del Sacro Cuore, Largo Francesco Vito 1, 00168 Rome, Italy; francesca.bardi97@gmail.com (F.B.); gianlu88us22@hotmail.it (G.B.); andreabrugnami@gmail.com (A.B.); greta.sfratta@hotmail.it (G.S.); delfina.janiri@unicatt.it (D.J.); gabriele.sani@unicatt.it (G.S.); 2 Department of Neuroscience, Section of Psychiatry, Fondazione Policlinico Universitario Agostino Gemelli IRCCS, Largo Agostino Gemelli 8, 00168 Rome, Italy; giorgio.kotzalidis@gmail.com (G.D.K.); alessio.simonetti@policlinicogemelli.it (A.S.); 3 Department of Psychiatry and Behavioral Sciences, Baylor College of Medicine, Houston, TX 77030, USA

**Keywords:** antipsychotic agents, oral, antipsychotic agents, long-acting injectable, treatment adherence, hospitalization, schizophrenia, relapse, suicide attempts

## Abstract

**Background/Objectives**: Long-acting injectable antipsychotics (LAIs) represent a significant advancement in the treatment of schizophrenia (SCZ), particularly for improving adherence and long-term outcomes. This study aimed to assess the clinical outcomes of patients receiving atypical LAIs compared to those on various oral antipsychotics over a one-year follow-up in a naturalistic setting. **Methods**: Sixty patients with SCZ were subdivided in two groups, those receiving LAIs (*n* = 25) and those receiving oral antipsychotics (*n* = 35). The groups were comparable for age, gender, educational attainment, employment status, marital status, smoking habits, and baseline SCZ severity, with no differences in baseline chlorpromazine equivalent dosages. **Results**: Over the follow-up period, patients in the LAI group discontinued treatment less frequently (*χ*^2^ = 4.72, *p* = 0.030), showed fewer suicide attempts (*χ*^2^ = 5.63, *p* = 0.018), fewer hospitalizations (*χ*^2^ = 4.95, *p* = 0.026), and fewer relapses (*χ*^2^ = 6.61, *p* = 0.010). Significant differences also emerged on the Drug Attitude Inventory (DAI-10) scores (F = 8.76, *p* = 0.005) and Body Mass Index (BMI) values (F = 8.32, *p* = 0.007), with the LAI group showing more favorable outcomes. **Conclusions**: LAIs, compared to oral antipsychotics, may promote treatment adherence, as shown by decreased hospitalization; furthermore, their use is related with better outcomes, like fewer relapses and less suicide attempts in individuals with SCZ in real-world settings.

## 1. Introduction

Schizophrenia (SCZ) is a severe and debilitating disorder that frequently follows a lifelong course [1]. Hallmarks of SCZ include recurrent relapses and deteriorating psychopathology and social functioning [2]. While some patients with SCZ recover well enough to achieve good daily functioning, take proper care of themselves, and have little or no symptoms, this subgroup is a tiny minority [3]. Naturalistic studies indicate that approximately 80% of patients experience relapse within a 5-year period [4,5], which ensues in negative consequences, regardless of illness stage [6]. Among these consequences, suicide is a leading cause of mortality among patients with SCZ [7], with an almost 3-fold higher risk during the first 5 years following their initial hospitalization [8].

Antipsychotic drugs (APs) are pivotal in the treatment of SCZ. APs have shown efficacy in reducing relapse rates in both first-episode and multiple-episode patients. The risk of relapse is reduced 2–6-fold when compared to the absence of AP therapy [4,9,10,11]. Compared to placebo or no treatment, APs are linked to lower all-cause mortality [12,13]. Despite their proven efficacy, adherence to AP treatment remains a major clinical challenge. Following first hospitalization, discontinuation rates escalate rapidly, from 36% within 30 days to nearly 50% within 60 days after discharge [14]. A subset of patients continues treatment with suboptimal adherence, with nearly half of them adhering to less than 70% of their prescribed oral medications [15]. Given the well-established link between non-adherence and risk of relapse (not to mention inefficacy), long-acting injectable antipsychotics (LAIs) have emerged as a strategy to enhance adherence and reduce relapse rates [16,17,18]. However, their use in everyday clinical practice is still limited, often being limited to patients with chronic SCZ with the aim to maintain adherence, while oral APs are still preferred during the early phases of the illness [19,20].

LAIs are delivered by healthcare professionals at regular intervals, thus ensuring that patients return to their scheduled visits; this facilitates treatment adherence. Switching from oral antipsychotics to LAIs seems an appropriate way to enhance adherence and thereby reduce the risk for relapse [21]. However, evidence on the comparative effectiveness of LAIs vs. oral formulations are inconsistent, with some studies endorsing their superiority over oral formulations [22,23], while others do not [24,25,26]. A 2021 meta-analysis comprising randomized controlled trials (RCTs), observational, and retrospective or prospective studies found LAIs to be linked with a lower risk of relapse and hospitalization [15]. An updated meta-analysis of 137 studies proved a small but consistent benefit of LAIs across RCTs, cohort, and retrospective or prospective studies [27]. Though discontinuation due to inefficacy or non-adherence was postponed, and hospitalization rates were lower in RCTs, a systematic review by the same group of investigators [28] confirmed a small benefit of LAIs in relapse prevention or all-cause discontinuation focusing on early-phase schizophrenia. This larger study indicated lower relapse risk in early illness, especially in newly diagnosed individuals [28]. Though hospitalization rates were comparable, a cluster-randomized trial found LAIs to be related to increased time to first hospitalization [29].

Long-acting injectable antipsychotics (LAIs) were first introduced in the mid-1960s as oil-based formulations of first-generation antipsychotics (FGAs). From the early 2000s, second-generation LAIs (SGAs) became available, based on aqueous formulations [30]. The SGAs approved for used in patients with SCZ and other disorders, including the psychotic spectrum and bipolar disorder, were intramuscular risperidone microspheres, paliperidone (9-hydroxyrisperidone) palmitate (PP), olanzapine pamoate, and aripiprazole monohydrate and dodecanoate (Lauroxil). The first risperidone LAI used microspheres, an innovative technology, but its use was limited by high costs and the need for injections every two weeks [31]. To overcome these limitations, risperidone’s drug manufacturers produced PP, a monthly formulation now also available as 3-month and 6-month injections; the last two are of doubtful cost-effectiveness [32]. Almost simultaneously with PP, olanzapine pamoate has been introduced, but its associated post-injection syndrome (sedation and confusion, and even delirium, which limit patient’s autonomy and are incompatible with driving back home for some time) [33] and the need to let the patient resting for 2–3 h at the outpatient unit or other healthcare facilities [34], limited its popularity among clinicians, not to mention the cardiometabolic side effects of this drug, which are similar to those of the oral formulation [35]. A 2013 US Food and Drug Administration (FDA) investigation [36], further added to the mistrust for this formulation. In 2013 and 2015, aripiprazole monohydrate and Lauroxil became available on the market, but only the former has received approval in the European Union [37]. More recently, risperidone LAI formulations were enriched by subcutaneous formulations [38], one of which showed good efficacy in once monthly and 2-month injections [39]. Such formulations have still to be introduced in Europe. In our service, we mostly use PP and aripiprazole monohydrate along of sorts of oral AP formulations.

LAIs have been compared with oral APs in terms of outcomes like relapses, rehospitalizations (length of hospital stay and number), and emergency room accesses. One study used a mirror design, i.e., one in which data in the same patient after the introduction of a LAI were compared to those before its introduction, when the patient was on oral APs, and found that the numbers of both emergency room accesses and hospitalization dropped after LAI prescription, as did the total time of hospital stay [40]. Another longitudinal study tested the FGA haloperidol oral vs. LAI in a randomized prospective 12-week study of patients with first-episode schizophrenia and found similar efficacy on the PANSS, but better Quality of Life (QoL), increased treatment adherence and less side effects [41]. However, the duration of this study was relatively limited. While the occurrence of less adverse events with LAIs compared oral might be surprising at a first glance, it was confirmed by two meta-analyses, although differences between molecules were recognized [42,43]; in contrast, another meta-analysis found more extrapyramidal and prolactin-related adverse events in LAIs compared to oral APs [44]. LAI APs were found to possess lower risk of all-cause and non-suicidal mortality in people with schizophrenia compared to oral APs, with better treatment adherence partly explaining this difference [45]. There is a dearth in the literature of comparisons of LAI AP to its own oral counterpart; only one study compared aripiprazole LAI to oral aripiprazole in acute episodes of psychosis in Chinese patients and found the two formulations to be comparable for efficacy and safety, but this study was a noninferiority study [46]. Summarizing the evidence, there is design heterogeneity and inconsistency of findings in LAI vs. oral AP comparisons [15,43].

Given the current uncertainty over the comparative efficacy of LAIs over oral antipsychotics, especially in naturalistic settings where real-world variables like treatment adherence and clinical complexity come into play, we aimed to assess the clinical outcomes of patients diagnosed with DSM-5 schizophrenia [47] receiving LAIs (i.e., aripiprazole or paliperidone) compared with those treated with oral APs, over a 12-month follow-up period in a naturalistic setting. This study compared treatment modalities rather than individual molecules or their respective formulations. The primary outcomes of interest were relapse, psychiatric hospitalization, suicidal ideation, treatment discontinuation, and BMI change. Our hypothesis was that LAIs would be associated with improved long-term outcomes compared to oral antipsychotic therapy.

## 2. Materials and Methods

### 2.1. Participants

Patients were recruited from the outpatient clinics of the Psychiatry Department of the Fondazione Policlinico Universitario Agostino Gemelli IRCCS in Rome, Italy, between January 2022 and December 2023. The study enrolled 60 patients diagnosed according to DSM-5 criteria with schizophrenia, multiple episodes, currently in acute episode [47]. Patients were treated either with long-acting injectable antipsychotics (LAIs; *n* = 25) or with second-generation oral antipsychotics (*n* = 35). The Structured Clinical Interview for DSM-5 Research Version (SCID-5-RV [48]) was used to diagnose SCZ and to identify psychiatric comorbidities. Additionally, we screened patients for Personality Disorders with the Structured Clinical Interview for DSM-5 Personality Disorders (SCID-5-PD; [49]). Diagnostic interviews were conducted by senior psychiatrists with acceptable inter-rater reliability (Cohen’s *κ* = 0.87).

Inclusion criteria required participants to meet the following: (a) ability of providing informed consent and comply with study procedures, which required basic literacy skills (corresponding to approximately 5 years of formal education) and fluency in Italian; (b) clinical diagnosis of SCZ according to DSM-5 criteria; (c) age between 18 and 65 years; (d) current treatment with either LAI or second-generation oral antipsychotic for at least three months prior to enrollment; (e) stable clinical condition at baseline, defined as absence of acute psychotic symptoms requiring hospitalization in the past 30 days. Exclusion criteria were: (a) intellectual disability or clinically significant cognitive impairment; (b) major medical or neurological conditions; (c) central nervous system infections; and (d) pregnant or breast-feeding women. Informed consent was obtained from all participants and their legal tutors before joining the study. The study adhered to the Principles of Human Rights as adopted by the 18th World Medical Association (WMA) General Assembly, Helsinki, Finland, June 1964 and amended by the 64th WMA General Assembly, Fortaleza, Brazil, October 2013; it obtained approval from the ethical committee of the Gemelli Foundation, ID 2014 Prot. N. 13864/18 of 21/02/2018.

### 2.2. Study Design

This was a naturalistic, longitudinal, observational cohort study conducted over a 24-month period with a 12-month follow-up for all patients.

Prior to baseline assessment, patients were non-randomly assigned to two groups. In one group (LAI group), patients were treated with paliperidone palmitate (*n* = 10) or aripiprazole monohydrate (*n* = 15). Patients in the oral AP group were treated with one of the following: olanzapine (*n* = 5), risperidone (*n* = 8), aripiprazole (*n* = 8), lurasidone (*n* = 4), clozapine (*n* = 7), and brexpiprazole (*n* = 3). Treatment allocation was based on the clinicians’ individualized judgment, without standardized protocols, and reflected routine clinical decision-making. This approach resulted in two groups that were comparable in key demographic and clinical characteristics, including symptom severity and chlorpromazine-equivalent dosage.

Assessments were conducted at baseline, after 6 months from initiating drug treatment, and after 12 months. At the baseline assessment, clinicians collected demographic and clinical variables on an appropriate socioepidemiological sheet. Demographic variables included age, gender, years of education, marital status, body mass index (BMI), and living status. Clinical variables included current psychopharmacological treatment, antipsychotic dosage standardized to chlorpromazine equivalents, lifetime suicidal ideation or behavior, and SCZ symptom severity. The evaluation of lifetime suicidal ideation was evaluated through the Columbia-Suicide Severity Rating Scale (C-SSRS) Lifetime version [50]. Psychopathological severity was assessed using the Positive and Negative Syndrome Scale (PANSS) [51].

Follow-up assessments were conducted at 6 and 12 months and included the following clinical outcomes: suicidal ideation or behavior, relapses, psychiatric hospitalizations, treatment discontinuation (drop-out), and BMI. Suicidal ideation or behavior was assessed using item 1 of the C-SSRS Since Last Visit version, which screens for the presence of any suicidal thoughts or behaviors. Relapse was defined as a ≥12-point increase in the PANSS total score, in line with evidence-based criteria proposed by Siafis and colleagues [52]. Drop-out was evaluated over a 1-year period from each patient’s initial visit and was defined as the occurrence of one or more of the following: (1) discontinuation of treatment for ≥5 weeks despite the psychiatrist’s prescription to maintain it; (2) failure to attend a scheduled psychiatric follow-up within one month of the appointed date; (3) a change in the antipsychotic agent prescribed; or (4) clinician’s decision. These different scenarios were grouped under a single “drop-out” category as they all represent indicators of treatment non-persistence, regardless of the underlying cause. Conversely, admission to a psychiatric ward was considered as indicative of treatment continuity.

At the 12-month follow-up, patients’ subjective attitude toward antipsychotic treatment was assessed using the 10-item Drug Attitude Inventory (DAI-10) [53]. This evaluation was conducted to explore patients’ personal perspectives on their ongoing pharmacological regimen and their overall engagement with treatment.

### 2.3. Assessment Tools

C-SSRS [50], PANSS [51], and DAI-10 [53] were used to assess suicidal ideation, severity of psychopathology, and subjective attitude toward antipsychotic treatment, respectively.

The Columbia-Suicide Severity Rating Scale (C-SSRS) [50] is a semi-structured clinical interview used to evaluate suicidal ideation and behavior. It includes multiple versions tailored to different time frames, including the “Lifetime” and the “Since Last Visit” versions, which were both used in this study. Total scores range from 1 (whish to be dead) to 5 (suicidal ideation with a plan and intent). Specifically, the C-SSRS begins by asking two questions that assess a participant’s desire to be dead (e.g., “I wish I was dead”) and nonspecific active suicidal thoughts (e.g., “I’ve thought about killing myself”). If the responses to both questions are negative or the answer to Question 1 is yes and Question 2 is no, the participant is considered not to have active suicidal ideation. A binary categorical variable was created based on the reported severity: nonsuicidal ideation (score of 0–1 points) and suicidal ideation (score of 2–5 points) [54]. The scale has been translated in various languages confirming its original good psychometric properties, including high inter-rater reliability (κ > 0.80) and predictive validity for suicidal behavior [55].

The Positive and Negative Syndrome Scale (PANSS) [51] is a clinician-administered instrument used to assess symptom severity in patients with SCZ. The scale comprises 30 items grouped into three subscales: Positive symptoms (7 items), Negative symptoms (7 items), and General Psychopathology (16 items). Each item is scored on a 7-point scale, ranging from 1 (absent) to 7 (extreme). Total scores range from 30 to 210, with higher scores indicating more severe psychopathology. The tool has demonstrated adequate internal consistency, external convergent and divergent validity and reliability in foreign language translations [56]; its validated Italian version that we used has demonstrated a 5-factor-structure, with good internal consistency (Cronbach’s α ranging from 0.73 to 0.83 across subscales) and strong inter-rater reliability (ICC > 0.80) [57].

The Drug Attitude Inventory (DAI-10) [53] is a self-report questionnaire used to evaluate the patient’s subjective experience and attitude toward antipsychotic medication. The instrument consists of 10 true/false items that capture beliefs, preferences, and perceived benefits or adverse effects associated with treatment. Positive total scores suggest a favorable attitude toward medication adherence, while negative scores may indicate poor insight or reluctance to continue pharmacotherapy. Its 10-item version has shown similar psychometric properties to the 30-item version, from which it was developed, showing good homogeneity and test–retest reliability [58]. The Italian version used in this study has shown acceptable internal consistency (Cronbach’s α = 0.84) [59].

### 2.4. Statistical Analyses

Descriptive statistics were computed for all variables. Between-group comparisons (patients treated with LAI vs. those receiving second-generation oral antipsychotics) were conducted using Pearson’s *chi*-square test (*χ*^2^) for categorical variables and one-way analysis of variance (ANOVA-1way) for continuous variables. When the assumption of homogeneity of variances was violated, Welch’s ANOVA was applied. For continuous variables we first tested their normality with the Shapiro–Wilk test [60] and then proceeded with parametric tests. For correlations we used Pearson’s *r* coefficient. No a priori power analysis was conducted, as the sample derived from naturalistic recruitment.

Categorical outcomes assessed at 6 and 12 months (i.e., suicidal ideation, relapse, psychiatric hospitalization, and treatment discontinuation) were analyzed using *chi*-square tests of independence. Continuous outcomes, including total scores on the DAI-10, PANSS subscales, and BMI, were compared between groups using ANOVA-1way or Welch’s ANOVA, as appropriate. Drop-outs were treated as a clinical outcome rather than missing data; therefore, no imputation was performed, and all patients were retained in the analyses accordingly.

All analyses were performed using the JASP statistical software (version 0.19.3, JASP Team, Amsterdam, The Netherlands) [61] and R (version 4.1.0, R Core Team) [62]. A two-tailed *p*-value of <0.05 was considered statistically significant.

## 3. Results

### 3.1. Demographics

The demographic and clinical characteristics of the sample are present in Table 1. The final sample included 60 patients divided into two groups, 25 receiving LAI and 35 receiving SGA APs. The two groups did not significantly differ in terms of age (*p* = 0.075), age at onset (*p* = 0.514), gender distribution (*p* = 0.965), years of education (*p* = 0.085), marital status (*p* = 0.706), and living status (*p* = 0.182). Baseline BMI (*p* = 0.080), smoking habits (*p* = 0.239), substance use (*p* = 0.426), chlorpromazine equivalents (*p* = 0.177), lifetime suicidal ideation (*p* = 0.064), PANSS-General (*p* = 0.479), -Positive (*p* = 0.064), and -Negative (*p* = 0.054) scale scores were also comparable.

### 3.2. Between-Group Comparisons of Clinical Outcomes

At 6 months, the between-group comparison of the relapse rate did not reach statistical significance (*p* = 0.080). At 12 months, the relapse rate was significantly lower in the LAI group (*n* = 2; 8.0%) compared to the Oral AP group (*n* = 11; 31.4%) (*χ*^2^ = 4.72, *p* = 0.030). Hospitalization rates at 6 months did not differ between groups (*p* = 0.133), while at 12 months, they were significantly reduced in the LAI group (*n* = 1; 4.0%) compared to the Oral AP group (*n* = 9; 25.7%) (*χ*^2^ = 4.95, *p* = 0.026). The prevalence of suicidal ideation or behavior during follow-up was significantly lower in the LAI group both at 6 months (*n* = 0 vs. *n* = 5; 0.0% vs. 14.3%; *p* = 0.048) and at 12 months (*n* = 2 vs. *n* = 13; 8.0% vs. 37.1%; *p* = 0.010). Drop-out rates were significantly lower in the LAI group at 6 months (*n* = 0; 0.0%) compared to the Oral AP group (*n* = 7; 20.0%) (*p* = 0.017), and at 12 months (*n* = 2 vs. *n* = 13; 8.0% vs. 37.1%; *χ*^2^ = 6.61, *p* = 0.010). DAI-10 scores were significantly higher in the LAI group than in the Oral AP group (F = 8.76; *p* = 0.005) at 12 months. Finally, at 12 months, the LAI group showed significantly lower BMI compared to the Oral AP group (F = 5.89; *p* = 0.019).

**Figure 1:**
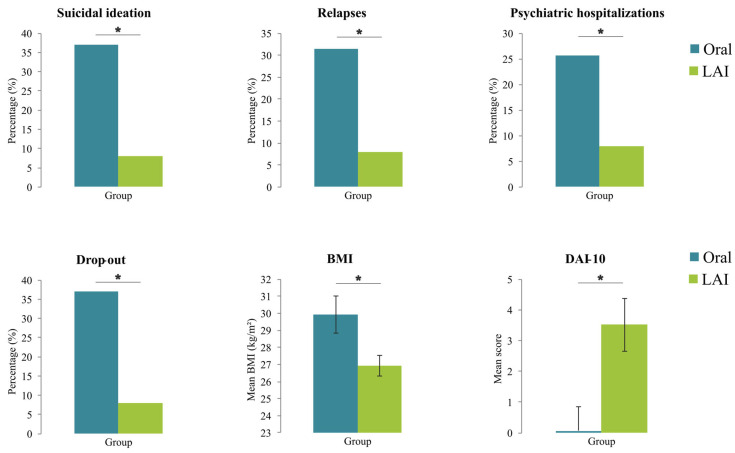
Between-group comparisons of clinical outcomes at the 1-year follow-up. Bar plots illustrate comparisons between Oral AP (gray-blue) and LAI groups (light green) across six clinical outcomes, i.e., suicidal ideation, relapse, psychiatric hospitalization, drop-out, BMI, and DAI-10. Values represent percentages or means. * indicates *p* < 0.05. Abbreviations: BMI, Body Mass Index; DAI-10, Drug Attitude Inventory-10 items; LAI, long-acting injectables antipsychotics; Oral, Oral Antipsychotics group.

The results of the between-group comparisons at the 1-year follow-up are summarized in Table 2 and visually represented in Figure 1.

## 4. Discussion

This naturalistic longitudinal cohort study compared the clinical outcomes of patients with SCZ treated with LAIs versus SGA oral APs over a 12-month period. The results of this study can be summarized as follows: at the 1-year follow-up, patients treated with LAIs were found to have significantly lower rates of relapse, hospitalization, and suicidal ideation, as well as reduced treatment discontinuation (a proxy of treatment compliance), a more favorable attitude toward pharmacotherapy (a proxy of treatment adherence), and a smaller increase in BMI compared to those receiving oral APs.

The current data suggest that LAI APs may be associated with greater therapeutic advantages than oral antipsychotic drugs in decreasing relapse rates and hospitalizations due to mental conditions over a long-term follow-up. The findings align with a significant corpus of observational and retrospective data endorsing the efficacy of LAIs in sustaining clinical stability and diminishing the risk of relapses and hospitalization in patients with schizophrenia [63,64,65,66,67,68,69]. Given their favorable impact on treatment adherence and relapse prevention, several authors have proposed the use of LAIs in the early phases of schizophrenia, arguing that early and sustained pharmacological intervention may lead to significantly improved long-term outcomes [15,70,71]. However, the literature remains divided on this topic. A meta-analysis by Kishimoto et al. [63], which included 21 RCTs, reported no significant superiority of LAIs over oral APs in relapse prevention, whether assessed at the longest follow-up point or at specific time intervals. In contrast, a later and larger meta-analysis by the same group [15], incorporating RCTs, observational studies, and mirror designs, found LAIs to be associated with a reduced risk of both relapse and hospitalization. The results of more recent experiments are more controversial. A cluster-randomized trial by Kane et al. [29] indicated no difference in overall hospitalization rates, despite a considerably prolonged duration to first hospitalization in patients administered LAIs. Conversely, research concentrating on early-phase schizophrenia has produced inconclusive findings, in that some studies have not shown distinct benefits of LAIs compared to oral formulations in decreasing relapse or hospitalization rates [24,25]. These differences are presumably due, at least in part, to methodological differences. Studies not demonstrating benefit were mostly RCTs, which, due to their methodological rigor, can lack external validity owing to the use of tightly defined patient groups and rigorously regulated procedures that may not accurately represent the intricacies of standard clinical practice, as is the case of our real-world study. In contrast, naturalistic studies include broader and more representative samples, with treatment delivered under real-world conditions. These differences count when meta-analyzing pooled data. Nonetheless, the absence of randomization in our study may have introduced some degree of confounding. Although groups were matched on key baseline variables, unmeasured factors might have influenced treatment allocation and outcomes. We acknowledge this as an inherent limitation of naturalistic designs, but also one that mirrors the complexity of real-world clinical decision-making and supports the relevance of observational data in complementing evidence from RCTs.

Our study also found that individuals receiving LAIs were less likely to report suicidal ideation at the long-term follow-up in comparison to those taking oral formulations. This is in line with previous extensive observational cohort studies and meta-analyses of RCTs, indicating that the administration of LAIs is correlated with reduced mortality compared to the use of oral antipsychotic medications [14,69,72,73]. Patients treated with LAIs had substantially reduced risks of suicide attempts, all-cause mortality, and natural-cause mortality, according to a large population-based cohort study that followed nearly 5000 individuals newly diagnosed with schizophrenia over a 16-year period [73] and in a pooled-data meta-analysis [45]. Although the high rate of mortality associated with schizophrenia is well established, little is known about what contributes to it. Several elements have been suggested as potential causes, including environmental factors, treatment non-adherence, insufficient medical comorbidity management, careless lifestyle, and the recurrence of psychotic episodes [74]. LAIs might contribute to mitigating these risk factors by promoting greater treatment continuity, reducing symptom relapse, and stabilizing illness trajectories [69,75,76]. The potential anti-suicidal effect of LAIs may therefore be mediated indirectly through improved adherence, reduced illness instability, and better control of affective and psychotic symptoms, domains closely linked to suicide risk in patients with schizophrenia, but also relevant across a variety of other psychiatric disorders [77,78,79]. However, lower rates of suicidal ideation in the LAI group may also be influenced by other unmeasured factors. For instance, variations in psychosocial support (e.g., familial engagement, availability of community resources, or involvement in psychosocial therapies) may have played a role in mitigating suicide risk. Likewise, the frequency of clinical follow-up, and the extent of residual symptom intensity, especially concerning emotional symptoms and hopelessness, may have influenced the observed outcomes. Subsequent research should incorporate these variables to thoroughly investigate the causes behind suicidal thoughts in this demographic.

In addition to clinical outcomes, our findings revealed a significantly more positive subjective attitude toward antipsychotic treatment, assessed through the DAI-10, among patients receiving LAIs compared to those on oral formulations. A favorable attitude toward medication is a known predictor of adherence and long-term treatment engagement, reflecting greater treatment satisfaction, insight, and perceived efficacy [80,81]. Moreover, patients in the LAI group exhibited a substantially lower rate of treatment discontinuation over the 12-month period, reinforcing the hypothesis that LAIs may be associated with improvements in objective dimensions of adherence via subjective perception [65,82,83]. Despite this evidence, the prescription of LAIs remains limited in routine clinical practice since many psychiatrists remain cautious toward their use [84]. Three main factors have been identified as contributors to this reluctance: limited availability of second-generation LAI options, patients’ rejection of LAIs, and their fear of relapses due to the switch [85]. Additional barriers include limited flexibility about dosage, low control of potential adverse effects, healthcare system constraints (e.g., reimbursement or accessibility), and the absence of strong guideline recommendations for their early use in first episodes of schizophrenia [86,87,88]. Our results challenge some of these assumptions by showing that, in a naturalistic setting, LAIs may support improved pharmacological adherence in objective terms but may also positively influence patients’ subjective experience of treatment, a widely recognized critical determinant of long-term therapeutic success [89]. Additionally, access to LAIs and clinical attitudes toward their use are also shaped by national healthcare systems. In some countries, limited reimbursement policies or administrative barriers may restrict availability, particularly in outpatient or community settings [90]. These systemic and regulatory factors should be considered when interpreting the underutilization of LAIs in clinical practice.

Beyond symptom control and relapse prevention, antipsychotic treatment in SCZ also carries significant implications for physical health, particularly regarding metabolic risk. Patients with schizophrenia face disproportionately high rates of metabolic syndrome, a cluster of conditions that includes obesity, dyslipidemia, hypertension, and type 2 diabetes, which contribute substantially to excess morbidity and mortality in this population [91,92]. In this context, our findings revealed a significantly lower BMI at 12-month follow-up in the group treated with LAI antipsychotics compared with those receiving oral formulations. Although previous studies have reported inconsistent differences in the overall metabolic risk profile between LAIs and oral antipsychotics [93,94], some metabolic parameters (e.g., total and low-density lipoprotein (LDL)-cholesterol, triglycerides, prolactin levels, waist circumference, and QTc interval) have been shown to be more favorable in patients receiving LAIs [95]. Importantly, the risk of metabolic side effects appears to be more closely related to the specific antipsychotic agent rather than the route or timing of administration. Agents such as clozapine, olanzapine, paliperidone, and quetiapine, whether oral or LAI, are consistently associated with greater risk of weight gain and metabolic disturbance [96,97,98,99,100]. Other studies are in line with our findings, suggesting that LAIs may be associated with less pronounced weight gain compared to oral antipsychotics, particularly when examining specific compounds. Evidence indicates that patients treated with oral aripiprazole tend to gain more weight, and at a faster rate, than those receiving its LAI equivalent [101,102]. More recent data from naturalistic studies with extended follow-up further support these results, highlighting a slower and less severe trajectory of weight gain in patients receiving LAI treatment [103]. The lower BMI observed in our LAI group may reflect not only differences in pharmacokinetic stability and medication exposure but also the indirect benefits of improved adherence and reduced relapse, since antipsychotic-induced weight gain is associated with increased nonadherence, discontinuations, dose escalations, polypharmacy, or emergency interventions, factors frequently associated with non-adherence to treatments [104,105]. This suggests that minimizing dose escalations and maintaining consistent treatment regimens, as facilitated by LAIs, could mitigate the metabolic side effects associated with antipsychotics medications.

LAIs are traditionally reserved for chronic, multiple-episode patients with poor compliance/adherence to treatment. Well, this is no longer sustainable. There is a need to deepen our understanding of neurodevelopmental trajectories in early-phase schizophrenia, particularly regarding treatment response and long-term outcomes, by investigating neurobiological underpinnings through neurophysiological markers [106,107]. Our sample was not composed of patients with chronic SCZ, but comprised first-episode patients as well. Young people with first-episode SCZ were shown to benefit from LAI AP treatment [87,108,109,110]. Hence, there is no reason for which they should be denied effective treatment timely and with no delay, since withholding treatment from a young patient with SCZ could worsen his/her long-term outcomes [111,112] in a period of brain maturation [113].

### Limitations

Some limitations must be acknowledged. First, the relatively small sample size (N = 60) limits the statistical power and generalizability of the findings. Second, the absence of randomization introduces a potential risk of selection bias, as treatment allocation was based on clinical judgment rather than random assignment; however, it also allowed for the formation of two groups that were homogeneous in key clinical and demographic characteristics at baseline. Third, while BMI was used as a proxy for metabolic side effects, we acknowledge that it does not fully capture the broader metabolic impact of antipsychotic treatment. Relevant parameters such as fasting glucose, lipid levels, or prolactin were not systematically collected, limiting the depth of our assessment. Similarly, suicidality at follow-up was assessed using item 1 of the C-SSRS (since last visit), which may not fully capture the complexity of suicidal risk. Fourth, the oral antipsychotic group included multiple molecules (e.g., clozapine, olanzapine, risperidone, lurasidone, aripiprazole, brexpiprazole), and we did not perform molecule-specific comparisons (e.g., oral vs. LAI aripiprazole), but it should be underlined that just one study did this [46]. This pharmacological heterogeneity could have introduced confounding effects, although we attempted to minimize this by standardizing doses using chlorpromazine equivalents. The ideal oral vs. LAI comparison would have limited the comparison of each LAI to its oral counterpart, but again, we could only compare aripiprazole monohydrate (*n* = 15) to oral aripiprazole (*n* = 8) and this would have needed non-parametric statistics that would be less powerful. Furthermore, current trends in aripiprazole LAI administration involve the now-approved two-injection start [114], which has shown pharmacokinetic advantages in patients with psychoses [115], but few of the 15 patients on aripiprazole LAI received the two-injection start, which needs no oral supplementation. Last, even if baseline characteristics were comparable between groups, unmeasured confounding factors, including outpatient care setting, may have influenced clinical outcomes and should be considered when interpreting the results. Despite these limitations, the strengths of this study include its naturalistic design, which enhances real-world applicability, the 12-month follow-up, and the use of validated assessment tools (C-SSRS, PANSS, DAI-10) that ensures reliable assessments of both symptom severity and patient-reported outcomes. Furthermore, we adhered to The Strengthening the Reporting of Observational Studies in Epidemiology (STROBE) Statement (Supplementary Table S1), which is a useful instrument for conducting and reporting adequate cohort studies [116].

## 5. Conclusions

This naturalistic longitudinal study provides further support for the clinical effectiveness of long-acting injectable antipsychotics in the treatment of schizophrenia. Importantly, our results suggest that long-acting injectable antipsychotics may be associated with indirect benefits for both psychiatric and physical health outcomes, possibly mediated by improved treatment adherence and greater symptom stability. Despite this, long-acting injectable antipsychotics remain underutilized, particularly in early-phases of schizophrenia, due to persistent clinical hesitancy and structural barriers. Our findings raise questions about this cautious approach and support consideration of a shift toward more proactive, personalized use of long-acting injectable antipsychotics, especially in patients with early signs of non-adherence or heightened risk of relapse and suicidality, common characteristics in patients diagnosed with schizophrenia.

## Figures and Tables

**Table 1:** Baseline sociodemographic and clinical characteristics of the sample.

Characteristic	Oral Antipsychotic (*n* = 35)	LAI (*n* = 25)	*p*-Value
Demographic characteristic			
Age (y), mean ± SD	36.86 ± 13.58	43.04 ± 12.60	0.075
Age at onset (y), mean ± SD	24.23 ± 10.42	22.84 ± 5.84	0.514
Gender, No. (%)			
Female	17 (48.6%)	12 (48.0%)	0.965
Male	18 (51.4%)	13 (52.0%)	
Education (y), mean ± SD	11.51 ± 2.90	13.16 ± 3.97	0.085
Marital status, No. (%)			
Not married	31 (87.5%)	21 (84.0%)	0.706
Married	4 (12.5%)	4 (16.0%)	
Living status, No. (%)			
Living alone	1 (3.0%)	3 (12.0%)	0.182
Living with someone	34 (97.0%)	22 (88.0%)	
Clinical characteristic			
BMI, mean ± SD	29.35 ± 5.45	26.78 ± 5.54	0.080
Smoking, No. (%)			
Yes	21 (61.0%)	10 (39%)	0.239
No	14 (39.0%)	15 (61.0%)	
Substance use, No. (%)			
Yes	16 (45.5%)	14 (56.0%)	0.426
No	19 (54.5%)	11 (44.0%)	
Chlorpromazine equivalent (mg/d), mean ± SD	307.14 ± 124.94	268.00 ± 96.70	0.177
Lifetime suicidal ideation, No. (%)			
Yes	15 (42.9%)	5 (20.0%)	0.064
No	20 (57.1%)	20 (80.0%)	
PANSS General, mean ± SD	47.16 ± 15.85	49.96 ± 13.79	0.479
PANSS Positive, mean ± SD	19.03 ± 12.32	24.48 ± 10.02	0.064
PANSS Negative, mean ± SD	20.46 ± 10.70	15.64 ± 8.29	0.054

Significance set at *p* < 0.05 (two-tailed). Abbreviations: BMI, body mass index; d, day; LAI(s), long-acting antipsychotic(s); mg, milligrams; *n* or No., number; PANSS, Positive And Negative Syndrome Scale; ± or SD, standard deviation; y, years.

**Table 2:** Between-group comparisons of clinical outcomes at 1-year follow-up.

Variables	Oral Antipsychotic (*n* = 35)	LAI (*n* = 25)	*χ2* or F	*p*-Value	Cramér’s V or *η^2^*
Suicidal ideation, No. (%)	13 (37.1%)	2 (8.0%)	6.61	**0.010**	0.29
Relapses, No. (%)	11 (31.4%)	2 (8.0%)	4.72	**0.030**	0.24
Psychiatric hospitalizations, No. (%)	9 (25.7%)	1 (4.0%)	4.95	**0.026**	0.24
Drop out, No. (%)	13 (37.1%)	2 (8.0%)	6.61	**0.010**	0.29
BMI, mean ± SD	29.93 ± 6.38	26.92 ± 3.07	5.89	**0.019**	0.09
DAI-10, mean ± SD	0.06 ± 4.72	3.52 ± 4.28	8.76	**0.005**	0.13

Significance set at *p* < 0.05 (two-tailed). Significant results are reported in bold. Abbreviations: BMI, body mass index; DAI-10, 10-item Drug Attitude Inventory; LAI(s), long-acting antipsychotic(s); *n* or No., number; SD, standard deviation.

## Data Availability

Data are available from the corresponding author upon reasonable request.

## Supplementary Materials

The following supporting information can be downloaded at https://www.mdpi.com/article/10.3390/healthcare13141709/s1, Supplementary Table S1. STROBE Statement CheckList for Bardi et al., Healthcare.

## Abbreviations

The following abbreviations are used in this manuscript:

AP(s)	Antipsychotic(s)
BMI	Body mass index
C-SSRS	Columbia Suicide Severity Rating Scale
DAI-10	10-item Drug Attitude Inventory
FDA	US Food and Drug Administration
FGA(s)	First-Generation Antipsychotic agent(s)
LAI	Long-Acting Injectable
QoL	Quality of Life
PANSS	Positive And Negative Syndrome Scale
SCZ	Schizophrenia
SGA(s)	Second-Generation Antipsychotic agent(s)
